# BAP1 mutations inhibit the NF-κB signaling pathway to induce an immunosuppressive microenvironment in uveal melanoma

**DOI:** 10.1186/s10020-023-00713-7

**Published:** 2023-09-14

**Authors:** Chao Zhang, Shuai Wu

**Affiliations:** 1 Department of Strabismus and Pediatric Ophthalmology, the Second Hospital of Jilin University, Changchun, 130041 P. R. China; 2 Department of Orbital Disease and Ocular Plastic Surgery, the Second Hospital of Jilin University, No. 218, Ziqiang Street, Nanguan District, Changchun, Jilin Province 130041 P. R. China

**Keywords:** BAP1, Gene mutation, NF-κB signaling pathway, Uveal melanoma, Tumor immune microenvironment

## Abstract

**Background:**

Tumor immune microenvironment regulates the growth and metastasis of uveal melanoma (UM). This study aims to reveal the possible molecular mechanism of BRCA1-associated protein 1 (BAP1) mutations in affecting the tumor immune microenvironment in UM through mediating the nuclear factor-κB (NF-κB) signaling pathway.

**Methods:**

TCGA and cBioPortal databases jointly analyzed the genes with high mutation frequency in UM samples. Following survival analysis of UM patients, UM samples with BAP1 mutations were subjected to immune cell infiltration analysis. The signaling pathways associated with the mutated genes were screened by GSEA. Subsequently, the differential BAP1 expression was analyzed in the selected UM cell lines with wild type (WT) or mutant type (MUT) BAP1.

**Results:**

Bioinformatics analysis identified 12 genes mutated in the UM samples, while only BAP1 mutations were related to the prognosis of UM patients. UM patients with BAP1 mutations had higher immune cell infiltration. BAP1 mutations inhibited the NF-κB signaling pathway, suppressing the cytokine secretion and antigen presentation by macrophages. Rescue experiments confirmed that overexpressed NF-κB could reverse the effect of BAP1 mutations on the immunosuppressive microenvironment, thus suppressing the malignant phenotypes of UM cells.

**Conclusion:**

BAP1 mutations may inhibit the NF-κB signaling pathway, repressing the cytokine secretion and antigen presentation by macrophages, which induces the immunosuppressive microenvironment, enhances the malignant phenotypes of UM cells and ultimately promotes the growth and metastasis of UM.

**Supplementary Information:**

The online version contains supplementary material available at 10.1186/s10020-023-00713-7.

## Introduction

Melanomas that occur at the choroid, ciliary body as well as iris of the eye are referred to as uveal melanoma (UM) (Chattopadhyay et al. 2016). UM originates from melanocytes in the stroma and presents the most prevalent primary intraocular tumor in the adult population (Jager et al. 2020; Smit et al. 2020; Rusnak et al. 2020). The prognosis of UM remains poor, with the lack of treatment options for patients developing metastasis; as an “immune-cold” tumor, UM has low mutational burden as well as special immunosuppressive microenvironment (Garcia-Mulero et al. 2021; Liau et al. 2023). The immunosuppressive microenvironment can sustain the development of UM (Meng et al. 2022). Despite the relatively lower mutational burden, underlying genetic aberrancies have been highlighted in UM, with gene mutations playing an important role in the oncogenesis and metastasis of UM (Smit et al. 2020). In this context, it is of significance to elucidate the possible molecular mechanism of gene mutations in the tumor immune microenvironment of UM.

BRCA1-associated protein 1 (BAP1), a ubiquitin carboxy-terminal hydrolase identified as a tumor suppressor, can regulate multiple processes including immune response; BAP1 mutations could lead to the occurrence of aggressive cancers such as UM (Louie and Kurzrock 2020). BAP1 has been suggested as a high-penetrance predisposition gene in UM (Read et al. 2016). Deficiency of BAP1 expression shared correlation with an immunosuppressive microenvironment in UM (Figueiredo et al. 2020). It should be noted that gene set enrichment analysis (GSEA) performed in the current study screened the nuclear factor-κB (NF-κB) signaling pathway as a key one regulated by BAP1 mutations in UM. Intriguingly, BAP1 was unfolded to increase the COX-2 and mPGES-1 expression by activating the NF-κB pathway (Viana et al. 2020). NF-κB is recognized as a crucial modulator of the development as well as the function of the immune system (Hayden and Ghosh 2011). As previously reported, NF-κB could be activated by NEMO, a gene found to be downregulated in UM cells (Singh et al. 2018). Notably, activation of the NF-κB signaling pathway could aid in regulating the tumor microenvironment in melanoma to suppress tumor growth (Liu et al. 2022). Therefore, these evidences allowed us to hypothesize in the current study that BAP1 mutations might regulate the tumor immune microenvironment to affect the growth and metastasis of UM, with the involvement of the NF-κB signaling pathway.

## Materials and methods

### Data collection and processing

Transcriptome, clinical information, and simple nucleotide variation data of UM patients were downloaded from the TCGA database, containing data of 80 μm patients. Data were processed using the Perl software.

### Mutated gene analysis

Mutated genes in UM patients were analyzed using the “maftools” package (Version 4.2) of the R language. The mutation types and mutation frequency of mutated genes were visualized, and the top 10 genes were selected to draw plots.

### Tumor mutational burden (TMB) analysis

Mutated genes in UM patients were analyzed using the “maftools” package (Version 4.2) of the R language, and the top 10 mutated genes were selected to draw waterfall plots.

### Correlation between clinicopathological features and TMB values in UM patients

The “limma” package of R language was used to analyze the correlation between the clinicopathological features and TMB values of UM patients. The main clinicopathological features of UM patients included TMN stage, disease grade, and gender. Statistical analysis was performed using Wilcox and Kruskal tests.

### Correlation between TMB values and the prognosis of UM patients

The correlation between TMB values and the prognosis of UM patients was analyzed using the “limma” and “survival” packages of R language. According to the median TMB value, UM patients were divided into low TMB group and high TMB group, and survival curves were plotted in years.

### Correlation between mutated genes and the prognosis of UM patients

The top 4 mutated genes based on mutation frequency were analyzed using the cBioPortal website, and the correlation of gene mutation with overall survival (OS) and progression-free survival (PFS) in UM patients was analyzed.

### Analysis of immune cell content and infiltration

Immune cell content in the samples was analyzed using the R language, and the data were visualized using pFilter = 0.05 as a filtering condition.

In addition, the UM samples with mutant type (MUT) or wild type (WT) BAP1 were used for immune cell infiltration analysis using the TIMER website. The main immune cells included cancer-associated fibroblasts, myeloiddendritic cells, macrophages, mast cells, natural killer (NK) cells, CD4^+^ T lymphocytes, CD8^+^ T lymphocytes, and B cells.

### Analysis of differentially expressed genes (DEGs) in UM samples with WT or MUT BAP1

DEGs in the UM samples were analyzed using the “limma” and “beeswarm” packages, including 54 μm samples with WT BAP1 and 26 μm samples with MUT BAP1.

### GSEA

“GSEA” software was used for “GO” and “KEGG” pathway enrichment analyses to screen BAP1 mutations-related signaling pathways.

### In vitro culture of UM cells

Three UM cell lines with WT BAP1 (Mel270, 92.1 and Mel290) and those with MUT BAP1 (MP65, MP46 and MP38) were selected for in vitro cell validation experiments. All cell lines were purchased from ATCC (Manassas, VA). Mel270, 92.1, and Mel290 cell lines were cultured in RPMI-1640 medium containing 10% FBS and 1% PS. MP cell lines were cultured in RPMI-1640 medium containing 20% FBS, 50 IU penicillin, and 50 µg/mL streptomycin.

### Sub-culture of macrophages

The human monocyte-macrophage THP-1 cell line was purchased from China Center for Type Culture Collection, Wuhan, China, and RPMI-1640 medium was used to adjust the concentration of the THP-1 cells to 1 × 10^8^ cells/mL. Next, the cells were seeded in the culture plates (1 mL/well) and cultured in an incubator with 5% CO2 at 37℃ for 2 h. The adherent cells were collected and cultured with 2 mL of RPMI-1640 medium containing serum and streptomycin in a 37℃ incubator. Culture medium was replaced every two days. When the cell density reached above 8 × 10^5^ cells/mL, sub-culture was performed. THP-1 was a suspension cell, which was directly sucked out, centrifuged and then passaged at a concentration of 1 × 10^5^ cells/mL. A total of 1 × 10^7^ cells were resuspended with 1 mL of FBS solution containing 10% DMSO and stored in a frozen storage tube in a -80℃ refrigerator overnight. The macrophage inducer PMA at a concentration of 130 ng/mL was used, with an initial cell number of 2 × 10^5^ cells/well. Cells were induced 24 h for subsequent Transwell co-culture assay.

### Co-culture of UM cells and macrophages

An in vitro cell co-culture system was established to partially simulate the tumor immune microenvironment, and the two groups of tumor cells and macrophages were placed at a 0.4 nm Transwell chamber in a ratio of 1:1, respectively. The UM cells were seeded in the apical chamber and activated tumor-associated macrophages THP-1 were seeded in the basolateral chamber. The cells were detached with trypsin and counted. Serum-free medium was added to adjust the cell concentration to 2 × 10^6^ cells/mL, and 100 µL cell suspension was seeded into the apical chamber. The cell concentration of macrophages in the basolateral chamber was 2 × 10^6^/mL, followed by culture in an incubator at 37℃ for 36 h. The cells in apical and basolateral chambers were both cultured using serum-free medium.

### NF-κB plasmid transfection

In the Rescue experiments, we constructed the NF-κB overexpression plasmid vector, and the CDS sequence for NF-κB is detailed in Table S1. A restriction site (BstBI/BamHI) was introduced to clone the universal biosynthetic CDS region gene fragments into the plvxpuro vector. The NF-κB-plvxpuro plasmid was used to transform E. coli DH5α competent cells, followed by overnight incubation on LB plates containing ampicillin. The extracted plasmid was verified by enzyme digestion and used for transfection.

### BAP1 nuclear staining

Immunocytochemistry was completed using previously published methods (Aughton et al. 2020; Kalirai et al. 2014). Cells in the slides were incubated overnight with 50–100 µL BAP1 primary antibody diluent (1:50 − 1:500; sc28383, Santa Cruz Biotechnology, Santa Cruz, CA) at 4℃ and added with ready-to-use secondary antibody dilution, followed by hematoxylin staining. The VECTASHIELD sealing medium (Vector Laboratories, Burlingame, CA) with DAPI was used to stain the nucleus. Fluorescent images were obtained under a Leica DMI3000 B inverted microscope (Leica Microsystems Inc., Buffalo Grove, IL).

### ELISA

The expression of IL-1β (E-EL-H0149c, Elabscience, Wuhan, China), MCP-1 (E-EL-H1159c, Elabscience), TGF-β (E-EL-H1587c, Elabscience) and CXCL-10 (E-EL-H0050c, Elabscience) were determined with the ELISA kits. Absorbance values were obtained at 450 nm using a microplate reader.

### RNA extraction and RT-qPCR

Total RNA was extracted using the EZBioscienceRNA kits (EZB-RN4, EZBioscience, USA) in accordance with the manufacturer’s guidelines. Next, the total RNA was reversely transcribed into cDNA using the RT reagent Kit (A0010CGQ, EZBioscience). Gene expression analysis was performed using RT-qPCR kits (A0012-R2, EZBioscience). With GAPDH as the internal reference, gene expression was quantified by 2^-ΔΔCt^ method. The list of primers is displayed in Table S2.

### Western blot

Protein lysates were generated using RIPA buffer with PMSF (P0013B, Beyotime, Shanghai, China) and protein concentrations were quantified by BCA Protein Assay (P0011/P0012, Beyotime). Lysates were loaded on SDS-PAGE gels for transfer to a PVDF membrane, followed by incubation with the primary antibodies (Table S3) and then with HRP-labeled rabbit anti-mouse IgG (ab6728, Abcam, Cambridge, UK; 1:2000-1:10000) or HRP-labeled goat anti-rabbit IgG (ab6721, Abcam, 1:5000); GAPDH (ab181602, Abcam,1:10000) served as the internal reference. Enhanced chemiluminescence (P0018M, Beyotime) was added, and an ImageQuantLAS4000C gel Imager (GE, USA) was used for development (Li et al. 2018).

### Flow cytometry

Cells to be tested were detached with trypsin and centrifuged at 800 rpm/min for 5 min. Next, cell precipitates were suspended in 100 µL of cell staining buffer, mixed with 5 µL of HumantrustainFCX, and blocked at room temperature for 10 min. FITC-anti-HLA-DR (MHCII) (307,620, BioLegend, San Diego, CA, ≤ 1.0 µg/million cells; ≤ 5µL/million cells) was added to incubate the cells on ice for 15 min. Following centrifugation at 350 g for 5 min and resuspension in 500 µL of cell staining buffer, MHCII expression was detected.

### CCK-8 and EdU assays

Cell proliferation was measured using the CCK-8 assay (C0037, Beyotime) according to the manufacturer’s instructions. Cells in the logarithmic phase were seeded into a 96-well plate with the density of 1 × 10^4^ cells/well, and 10 µL of CCK-8 solution was added to each well. After 2 h of incubation at 37℃, the absorbance of each sample was measured at 450 nm using a microtiter plate reader. Measurements were performed at 0, 24, 48, and 72 h, respectively.

Cells were seeded into a 96-well plate with the density of 2 × 10^6^ cells/well and cultured overnight. Next, 100 µL of 50 µM DMEM-diluted EdU solution was added to each well for incubation. The cells were fixed with 4% paraformaldehyde for 15 min, rinsed with 2 mg/mL glycine solution and incubated with 0.5% Triton-100 for 10 min, followed by staining with Apollo solution and with Hoechst reaction solution each for 30 min.

### Scratch assay

UM cells with MUT or WT BAP1 were seeded into 6-well plates with the density of 5 × 10^5^ cells per well. After scratches were made with a 10 µL pipette, the cells were cultured with serum-free medium in an incubator with 5% CO_2_ at 37℃. An inverted microscope was utilized to photograph the scratches of each well at 0 and 24 h, and the images were analyzed using the ImageJ software.

### Transwell assay

The Transwell apical chamber was coated with Matrigel for 30 min at 37℃. Cells were cultured in serum-free medium for 12 h and resuspended with serum-free medium (1 × 10^5^ cells/mL). Next, the medium supplemented with 10% FBS was added to the basolateral chamber. A total of 100 µL of cell suspension was added to the Transwell chamber, followed by fixation with 100% methanol and staining with 1% toluidine blue (Sigma-Aldrich, St. Louis, MO). Finally, five random regions were photographed under an inverted microscope and the cells were counted.

### Statistical analysis

The statistical analysis of the data in this study was performed with the use of the GraphPadPrism8.0 statistical software (Version 8.0.2, GraphPadSoftware, San Diego, CA). Numerical variable data obeying normal distribution are expressed as mean ± standard deviation, while those not obeying normal distribution as median ± interquartile range. The comparisons between two groups of independent samples were conducted by *t* test (obeying normal distribution) or Wicoxon rank sum test (not obeying normal distribution), and those among multiple groups of independent samples by one-way ANOVA (obeying normal distribution) or Kruskal Wallis test (not obeying normal distribution). Kaplan-Meier method and Log-rank test were used for survival analysis. All experiments were repeated at least 3 times. The tests were two-tailed tests and the test criterion was ɑ = 0.05.

## Results

### Joint analysis of TCGA and cBioPortal databases screened the top 10 genes in UM samples based on mutation frequency

**Fig. 1:**
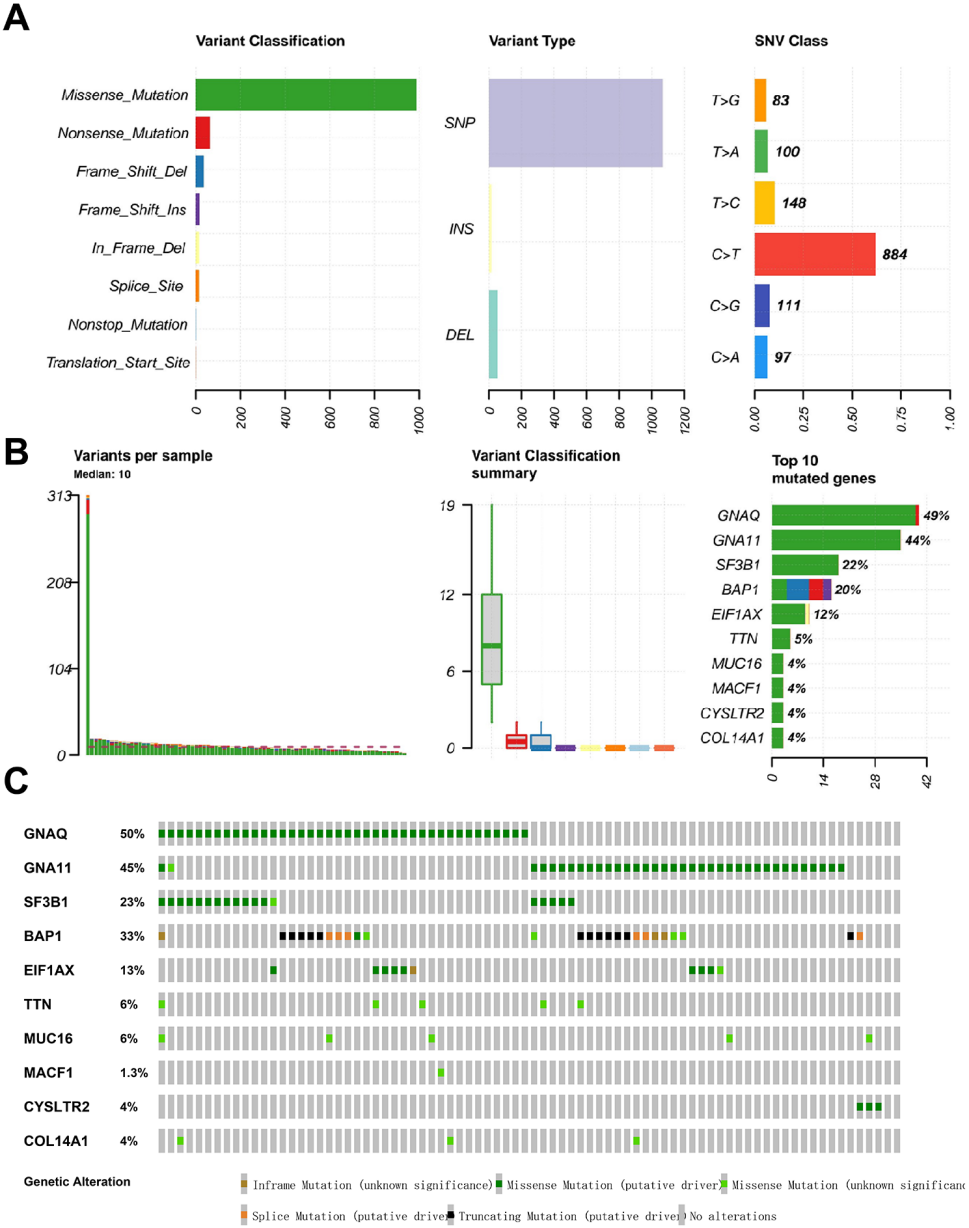
Joint analysis using the TCGA and cBioPortal databases to screen the mutated genes in the UM samples. **A**, The main mutation types in UM samples and the main types of base substitutions in single nucleotide variation data. **B**, The frequency of various mutations in UM samples and the top 10 genes with mutation frequency. **C**, cBioPortal-based analysis and verification of mutated genes

In order to explore the mutation genes playing roles in the UM, we first analyzed the SNP data from the TCGA database. The results determined the main mutation types and base substitution types (Fig. 1A): the mutation types were mainly missense mutations, followed by nonsense mutations, frameshift mutations, in-frame deletion and shear mutations, etc. Base substitution types were mainly C > T.

The top 10 genes based on mutation frequency (Fig. 1B) were then obtained, namely, GNAQ (49%), GNA11 (44%), SF3B1 (22%), BAP1 (20%), EIF1AX (12%), TTN (5%), MUC16 (4%), MACF1 (4%), CYSLTR2 (4%) and COL14A1 (4%). We used cBioPortal to analyze and verify the mutations of genes in UM, the results of which were consistent with those of the TCGA database (Fig. 1C).

### Clinicopathological features and prognosis of UM patients were not significantly associated with TMB values

**Fig. 2:**
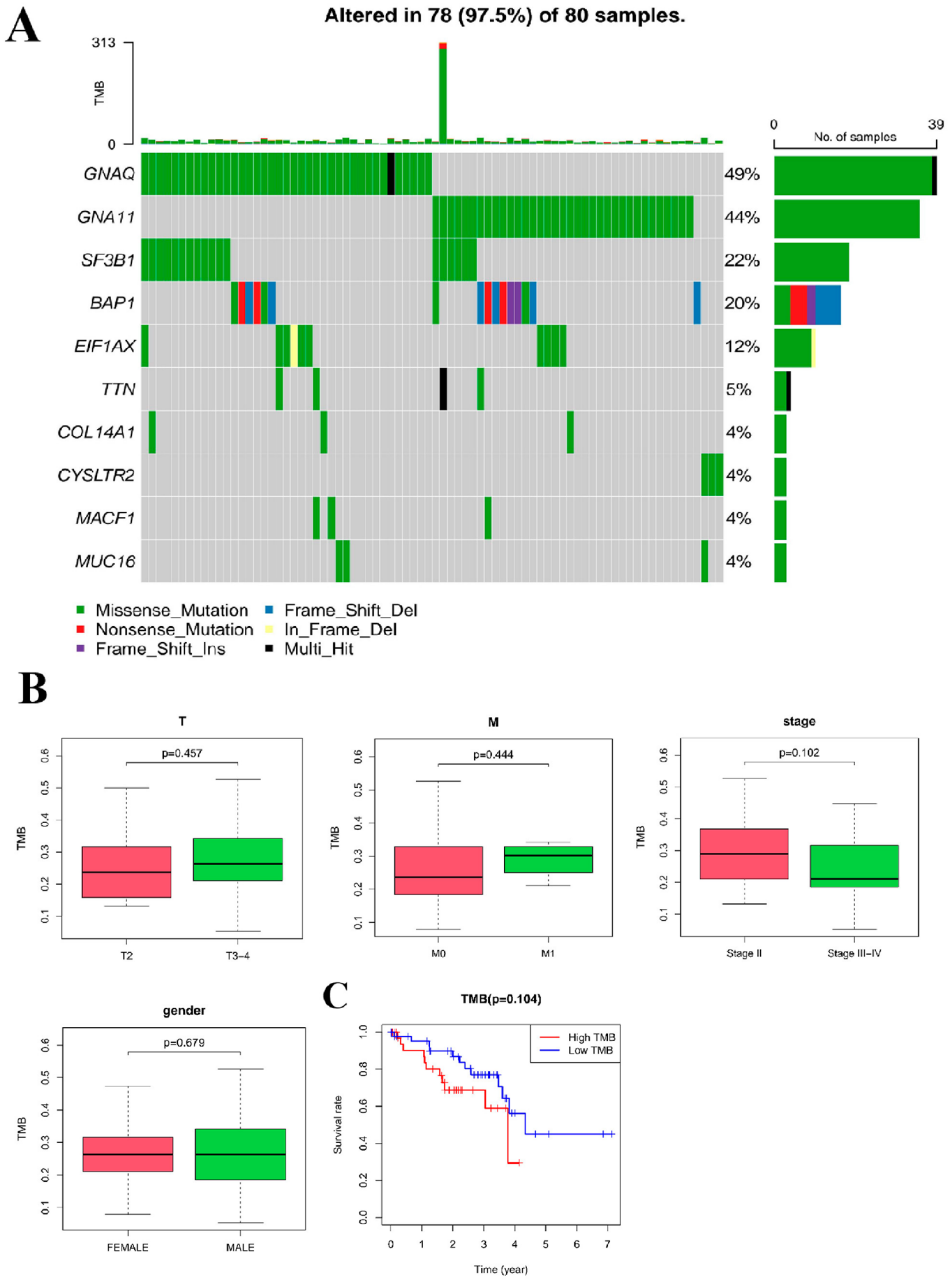
Correlation analysis between TMB values and clinicopathological characteristics and prognosis of UM patients. **A**, Mutation types and frequency of mutated genes in UM samples, and TMB value of each sample. The green region indicates the missense mutations, blue region indicates the frameshift deletion mutations, the red region indicates the nonsense mutations, the yellow region indicates the in-frame deletion mutations, the purple region indicates the frameshift insertion mutation, the black region indicates multiple mutations and the gray region indicates no mutations. **B**, Correlation between clinicopathological characteristics (clinical stage, grade and gender) and TMB value of UM patients. **C**, Survival analysis of UM patients in the high TMB group and the low TMB group. The red line indicates the UM patients in the high TMB group, and the blue line represents the UM patients in the low TMB group

To explore the relationship between mutated genes and TMB values, we first calculated the probability of gene mutation and TMB values in 80 μm patients. Our analysis results found that 97.5% of the UM samples had gene mutations, and they all showed low TMB values (Fig. 2A).

Next, we further explored the correlation between the clinicopathological features (TMN stage, clinical grade, and gender) and the TMB values of the UM patients. Our analysis suggested that none of these clinicopathological features were significantly associated with the TMB values (Fig. 2B). Finally, we found no statistically significant difference in survival of UM patients between those with low and high TMB values (Fig. 2C). The above results indicated that the clinicopathological characteristics and prognosis of UM patients were not significantly associated with TMB values, and that TMB values are not an independent factor affecting the survival and prognosis of UM patients.

### BAP1 mutations was closely associated with OS and PFS in UM patients

**Fig. 3:**
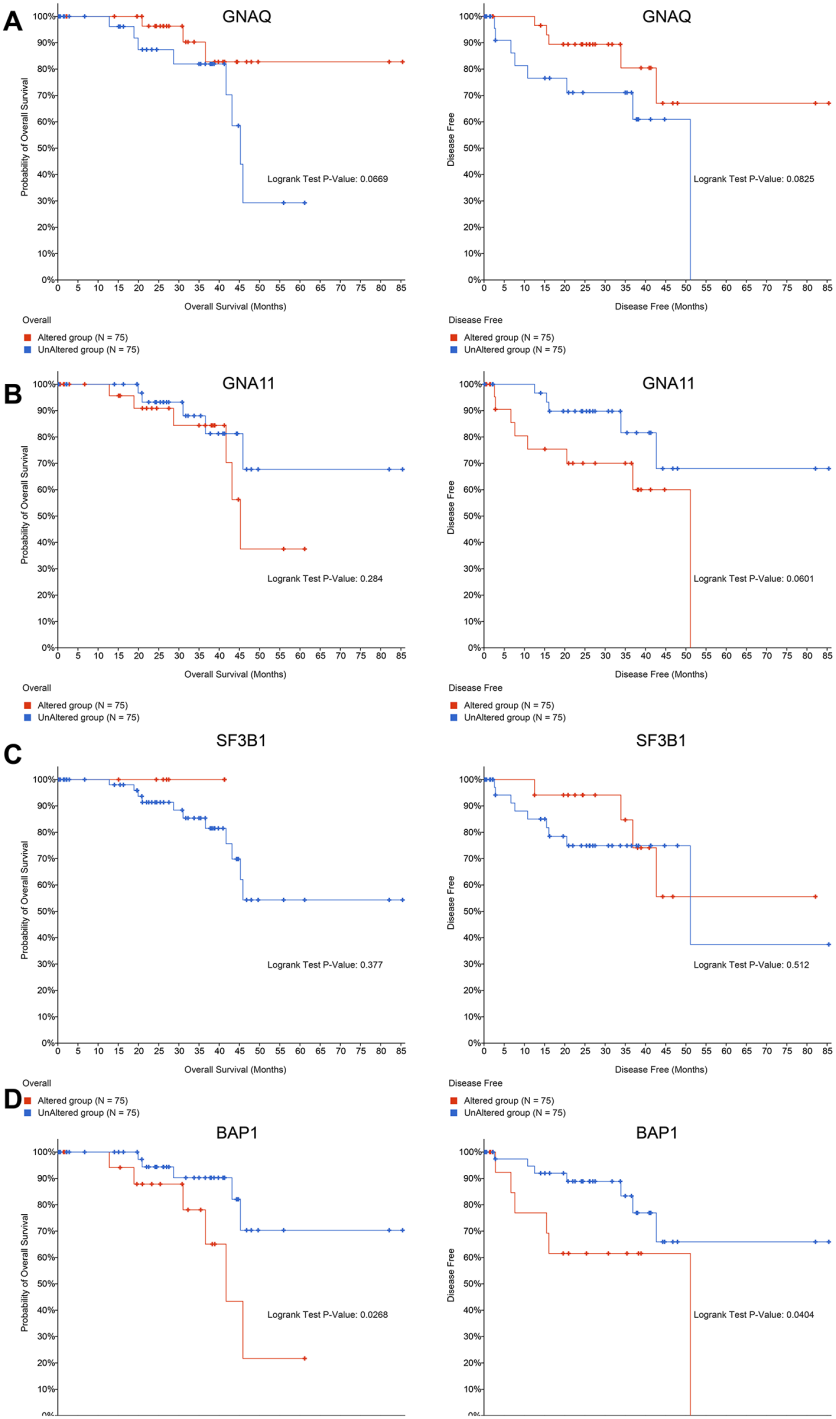
cBioPortal analysis of the correlation between the top 4 genes based on mutation frequency with OS and PFS in UM patients. Analysis of the OS and PFS of the mutated genes GNAQ **(A)**, GNA11 **(B)**, SF3B1 **(C)** and BAP1 **(D)**. Altered group: samples with gene mutation; unaltered group: samples without gene mutation

To identify the mutated genes that play key roles in UM samples, we used cBioPortal to analyze the correlation between the top 4 genes with mutation frequency (GNAQ, GNA11, SF3B1, and BAP1) and OS and PFS in UM patients. As shown in Fig. 3, only the BAP1 mutations were associated with OS and PFS in UM patients, and the remaining three mutated genes showed no significant association. Furthermore, patients with MUT BAP1 had lower OS and PFS as compared to those with WT BAP1.

### Immune cell infiltration was higher in UM samples with BAP1 mutations

**Fig. 4:**
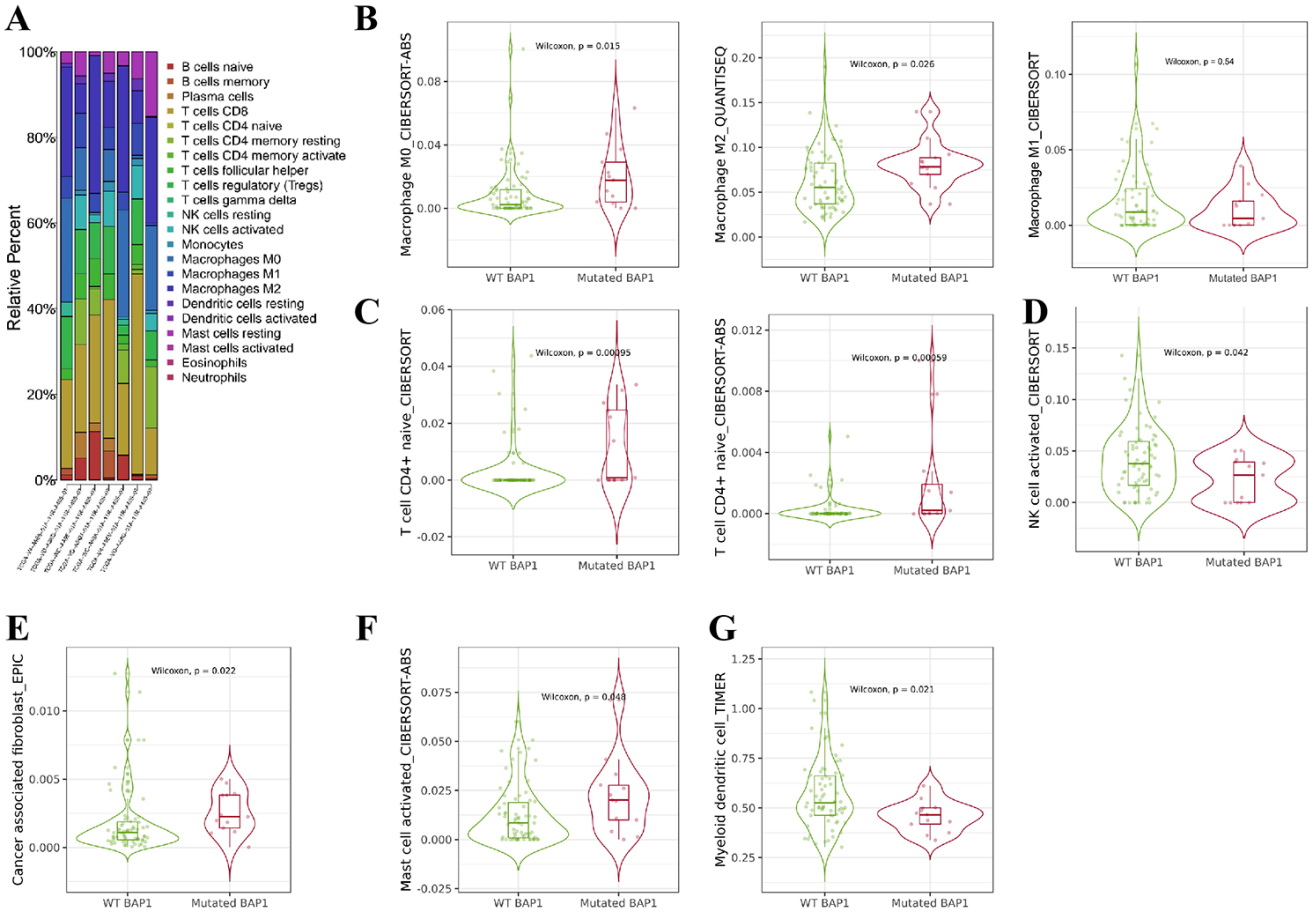
Analysis of immune cell content and immune cell infiltration in UM samples with BAP1 mutations. **(A)** Analysis of immune cell content of UM samples. B-G, Immune cell infiltration analysis of UM samples using the TIMER website to detect the infiltration of macrophages **(B)**, CD4^+^ T lymphocytes **(C)**, NK cells **(D)**, tumor-associated fibroblasts **(E)**, mast cells **(F)** and myeloid dendritic cells **(G)** in UM samples with WT or MUT BAP1, respectively. WT, wild type; mutated BAP1, mutant type BAP1.

Next, we analyzed UM samples in R language and found various immune cells expressed in UM samples, with the highest content of T cells, NK cells and macrophages (Fig. 4A).

We used the TIMER website to analyze the immune cell infiltration in the UM samples with WT and MUT BAP1. The results showed obvious differences in immune cell infiltration between the two sample groups. In contrast to the UM samples with WT BAP1, macrophages, CD4^+^ T lymphocytes, CD8^+^ T lymphocytes, tumor-associated fibroblasts, and mast cells had higher infiltration in the UM samples with MUT BAP1 (Fig. 4B-G).

### BAP1 mutations might regulate the NF-κB signaling pathway to affect cytokine secretion by macrophage and its antigen-presenting capacity

**Fig. 5:**
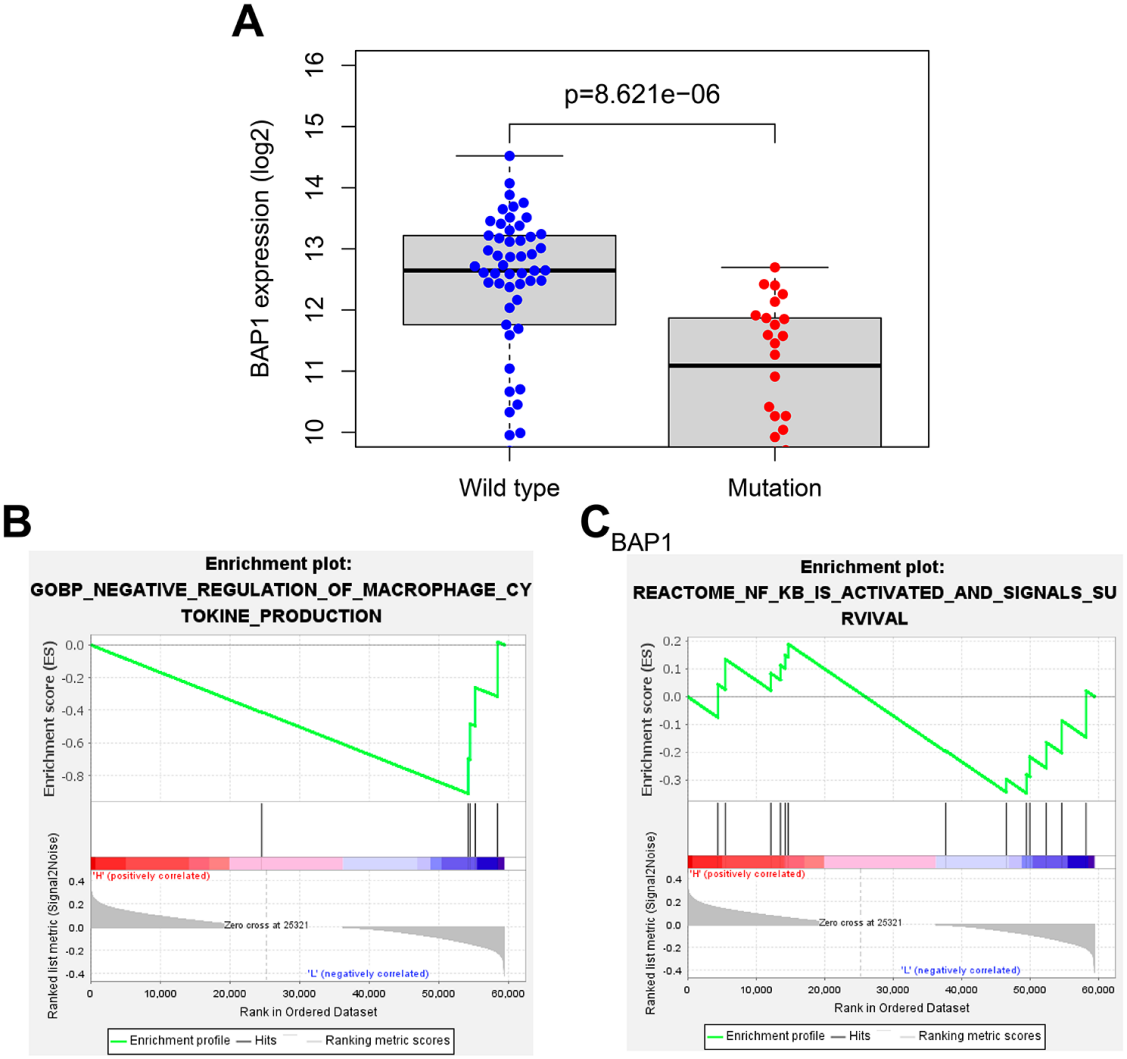
GSEA of the signaling pathways associated with the BAP1 mutations. **A**, Comparison of BAP1 expression levels in wild-type and mutant UM samples, with Wildtype indicating the wild-type group and Mutation indicating the mutant group; **B**, GSEA enrichment analysis of the negative regulation of macrophage cytokine production in the BAP1 high expression group and BAP1 low expression group; **C**, GSEA enrichment analysis of the NF-κB signaling pathway in the BAP1 high expression group and BAP1 low expression group

We further explored the effect of BAP1 mutations on BAP1 gene expression. As shown in Fig. 5A, when the mutation occurred, the BAP1 mRNA expression in the UM samples with MUT BAP1 was significantly reduced compared with that in the UM samples with WT BAP1.

The Gene Set Enrichment Analysis (GSEA) is suitable for comparing two different biological states, referred to as the treatment group and the control group, in order to determine which group of genes exhibits expression patterns that are more closely related to a specific biological process or pathway. This enables the inference of the role of target genes in contributing to this biological process. GSEA provides more reliable and highly credible results. To investigate the signaling pathways associated with BAP1 gene mutations, we divided UM samples into two groups based on the median expression level of BAP1. Subsequently, we performed GSEA enrichment analysis for “GO” and “KEGG” relevant signaling pathways as shown in Fig. 5B-C. The results revealed significant differences between the BAP1 high-expression group and the BAP1 low-expression group in the negative regulation of macrophage cytokine production and the NF-κB signaling pathway. Interestingly, the peak activity for both pathways was observed in the BAP1 low-expression group, indicating that these pathways are more active in the BAP1 low-expression group, particularly concerning the negative regulation of macrophage cytokine production and the NF-κB signaling pathway.

Previous study has reported that macrophages mainly secrete IL-1, and this molecule acts on macrophages in an autocrine manner, inducing macrophages to upregulate MHC-II, which is mainly related to innate immunity and affects a wide range of inflammation and immune responses (Tseng et al. 2022). In addition, the cytokines secreted by macrophages can further promote the transcription of related immune regulators by regulating the NF-κB signaling pathway (Diep et al. 2022). Based on the above results, we speculate that BAP1 gene mutations may affect the production and antigen presentation ability of macrophage cytokines by regulating the NF-κB signaling pathway, thereby participating in the development and progression of UM.

### Mel 270 and MP 46 cell lines were selected for subsequent experimentation

**Fig. 6:**
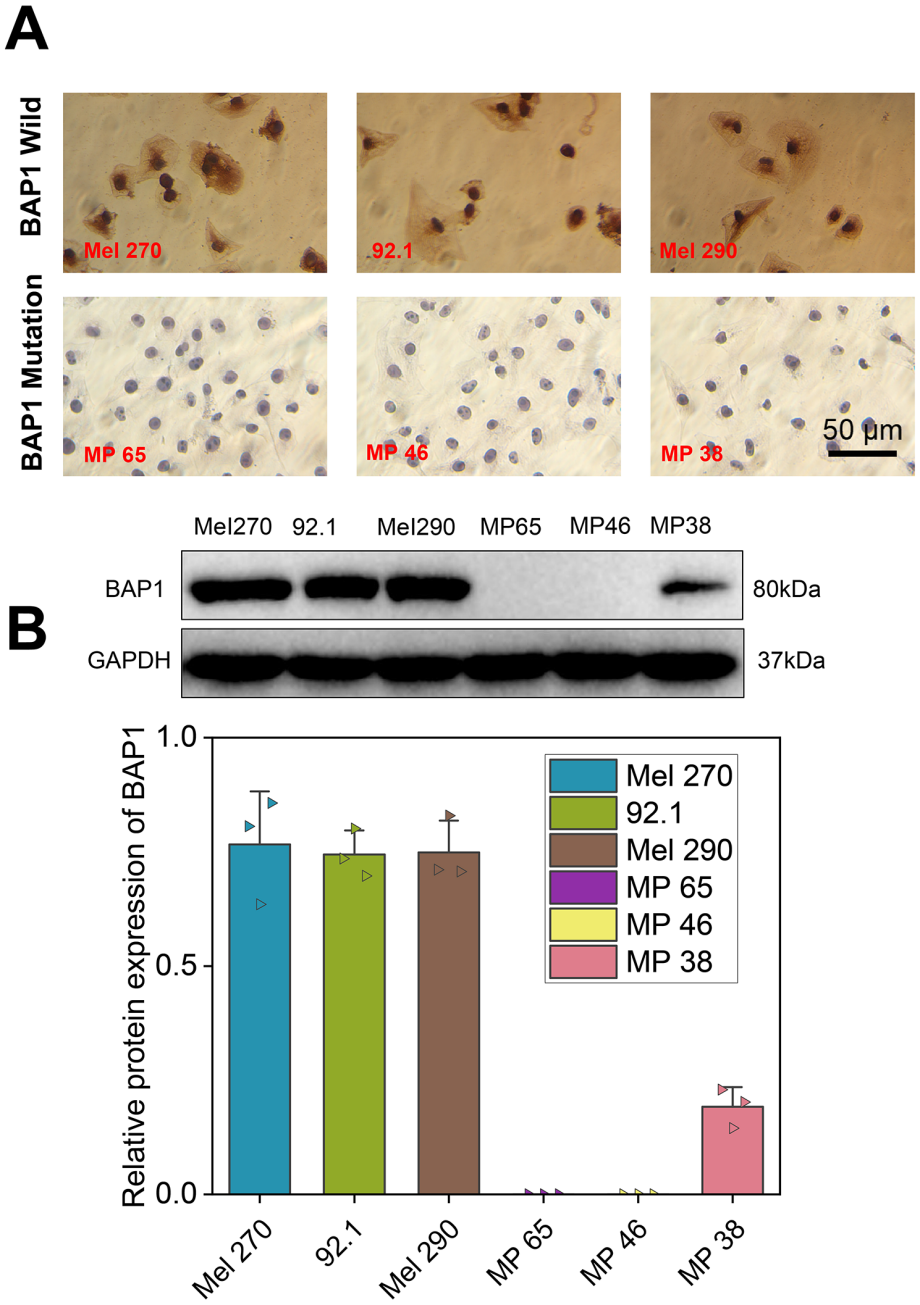
BAP1 mutation validation and cell line screening. **A**, Nuclear staining for BAP1 in the cell lines with WT or MUT BAP1. **B**, Western blot of BAP1 protein expression in the cell lines with WT or MUT BAP1. Cell lines with WT BAP1: Mel 270, 92.1, and Mel 290; cell lines with MUT BAP1: MP 65, MP 46 and MP 38

Three different cell lines with WT BAP1 and three with MUT BAP1, namely Mel 270, 92.1, Mel 290, MP 65, MP 46, and MP 38, were initially selected. According to previous literature, immunocytochemistry for nuclear BAP1 is the best way to identify gene mutation; in case of BAP1 mutations, the nuclear staining of BAP1 is lost (Farquhar et al. 2018). As shown in Fig. 6A, all of the cell lines with MUT BAP1 showed a loss of BAP1 nuclear staining, whereas BAP1 nuclear staining was observed in the cell lines with WT BAP1.

In addition, the BAP1 mutations are mostly inactivating mutations that affect the protein expression of BAP1 (Han et al. 2021; Kaler et al. 2022). Therefore, we further detected the BAP1 protein expression in each cell line by Western Blot. BAP1 protein expression was found in all cell lines with WT BAP1 and in the MP 38 cell line with MUT BAP1, but not in the other cell lines with MUT BAP1 (Fig. 6B).

Accordingly, we selected Mel 270 and MP 46 cell lines for subsequent experimentation.

### BAP1 mutations inhibited cytokine secretion and antigen presentation by macrophages

**Fig. 7:**
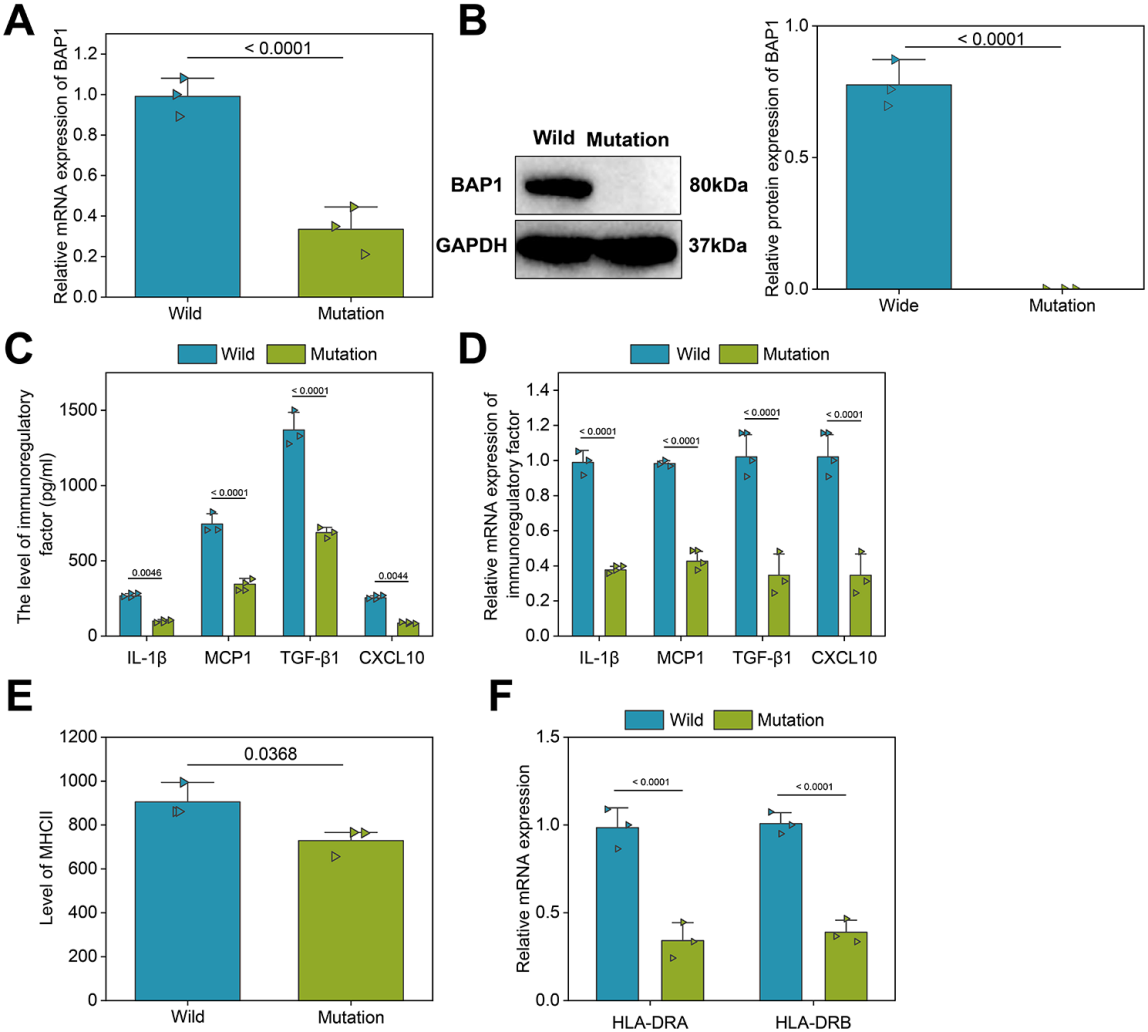
Effects of BAP1 mutations on the tumor immune microenvironment. **A**, The mRNA expression of BAP1 in the UM cells with WT or MUT BAP1 measured by RT-qPCR. **B**, The protein expression of BAP1 in the UM cells with WT or MUT BAP1 measured by Western Blot. **C**, ELISA detection of the expression of immune regulators (IL-1β, MCP1, TGF-β1 and CXCL10) in the in vitro co-culture system. **D**, RT-qPCR detection of the expression of macrophage-secreted cytokines (IL-1β, MCP1, TGF-β1 and CXCL10) after co-culture of UM cells with WT or MUT BAP1 and macrophages. **E**, MHCII expression on the macrophage surface by flow cytometry. **F**, The mRNA expression of MHCII alleles HLA-DRA and HLA-DRB on the surface of macrophages detected by RT-qPCR. WT, wild type; mutated BAP1, mutant type BAP1. * *p* < 0.05

First, we compared the mRNA and protein expression of BAP1 between wild-type and mutant UM cells. The results indicated significant differences in the expression levels of both mRNA and protein between the two groups, with BAP1 mRNA and protein expression markedly lower in the BAP1 mutant group compared to the wild-type group (Fig. 7A, B).

Next, to investigate the impact of BAP1 gene mutations on the immune microenvironment of UM, we conducted an in vitro co-culture experiment using wild-type and mutant BAP1 UM cells with THP-1 macrophages. The experimental results revealed that co-culture with mutant BAP1 UM cells led to downregulation of the protein and mRNA levels of IL-1β, MCP-1, TGF-β1, and CXCL-10 in THP-1 macrophages compared to co-culture with wild-type BAP1 UM cells (Fig. 7C, D).

Furthermore, we performed flow cytometry and RT-qPCR to examine the expression of MHCII, HLA-DRA, and HLA-DRB genes on the surface of macrophages co-cultured with UM cell lines. The results showed a significant decrease in the expression of MHCII, HLA-DRA, and HLA-DRB genes on the surface of THP-1 macrophages co-cultured with mutant BAP1 UM cells compared to those co-cultured with wild-type BAP1 UM cells, suggesting a decrease in the antigen-presenting capacity of macrophages (Fig. 7E, F).

These findings collectively demonstrate that BAP1 mutations can inhibit the secretion of cytokines by macrophages as well as their antigen-presenting abilities.

### Overexpression of NF-κB reversed the inhibitory effect of BAP1 mutations on the cytokine secretion and antigen presentation by macrophages

**Fig. 8:**
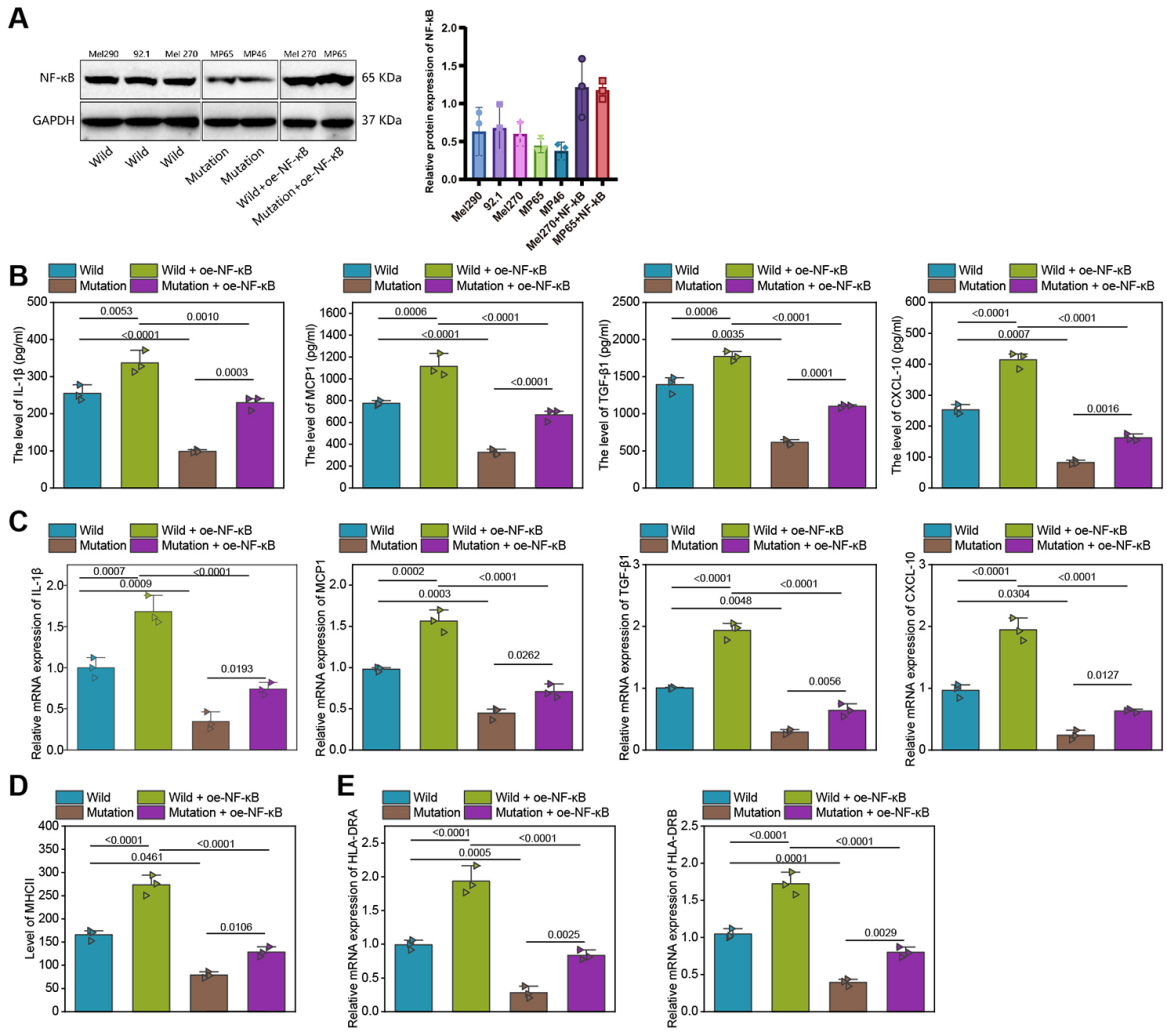
In vitro rescue experiments for verification of the effects of the BAP1 mutations on the tumor immune microenvironment in UM by regulating the NF-κB signaling pathway. **A**, Western blot detection of the expression difference of NF-κB between the UM cells with WT BAP1 and those with MUT BAP1. **B**, ELISA detection of the expression difference of immune regulators between the UM cells with WT BAP1 and those with MUT BAP1 after NF-κB overexpression. **C**, RT-qPCR detection of the expression difference of immune regulators between the UM cells with WT BAP1 and those with MUT BAP1 after NF-κB overexpression. **D**, Flow cytometry for the expression difference of MHCII between the UM cells with WT BAP1 and those with MUT BAP1 after NF-κB overexpression. **E**, The mRNA expression of the MHCII alleles HLA-DRA and HLA-DRB on the surface of macrophages as detected by RT-qPCR after NF-κB overexpression. WT, wild type; mutated BAP1, mutant type BAP1; WT + oe-NF-κB, UM cells with WT BAP1 and NF-κB overexpression; MUT + oe-NF-κB, UM cells with MUT BAP1 and NF-κB overexpression. * *p* < 0.05

We further validated the molecular mechanisms of the BAP1 gene mutation in regulating the NF-κB signaling pathway and influencing the immune microenvironment of UM through in vitro rescue experiments. Initially, we examined the expression of NF-κB protein in BAP1 wild-type and mutant UM cells using Western Blot. The results revealed lower expression of NF-κB in BAP1 mutant UM cells (Fig. 8A).

Next, we co-cultured THP-1 macrophages with mutant BAP1 cell line MP46, wherein NF-κB was overexpressed. We investigated the changes in secretion of macrophage factors and antigen presentation capabilities. The expression of immune regulatory factors in macrophages from each group was analyzed using ELISA and RT-qPCR. Our findings showed that the expression levels of IL-1β, MCP1, TGF-β1, and CXCL-10 in THP-1 macrophages co-cultured with BAP1 mutant UM cells were lower compared to those co-cultured with BAP1 wild-type UM cells. However, the levels of these immune regulatory factors increased when NF-κB was overexpressed in BAP1 mutant UM cells and co-cultured with THP-1 macrophages. Nevertheless, even in the presence of NF-κB overexpression, the expression of IL-1β, MCP1, TGF-β1, and CXCL-10 in THP-1 macrophages co-cultured with BAP1 mutant UM cells remained lower than those co-cultured with wild-type BAP1 UM cells (Fig. 8B, C).

Furthermore, flow cytometry and RT-qPCR were utilized to determine the expression levels of MHCII (an antigen presentation marker) and the mRNA levels of HLA-DRA and HLA-DRB, which are MHCII alleles, in THP-1 macrophages co-cultured with different groups of UM cells. The results indicated that compared to THP-1 macrophages co-cultured with BAP1 wild-type UM cells, those co-cultured with BAP1 mutant UM cells displayed lower levels of MHCII expression, as well as lower levels of HLA-DRA and HLA-DRB mRNA. When NF-κB was overexpressed in BAP1 mutant UM cells and co-cultured with THP-1 macrophages, the expression of MHCII and mRNA levels of HLA-DRA and HLA-DRB in THP-1 macrophages were higher than when co-cultured with BAP1 mutant UM cells. Additionally, when co-cultured with THP-1 macrophages, both the expression of MHCII and the mRNA levels of HLA-DRA and HLA-DRB in THP-1 macrophages were lower when NF-κB was overexpressed in BAP1 mutant UM cells compared to when co-cultured with BAP1 wild-type UM cells (Fig. 8D-E).

The above results suggest that upregulating the NF-κB signaling pathway can partially restore the decreased secretion of cytokines and reduced antigen presentation caused by BAP1 mutations.

### BAP1 mutations enhanced the malignant phenotypes of UM cells

**Fig. 9:**
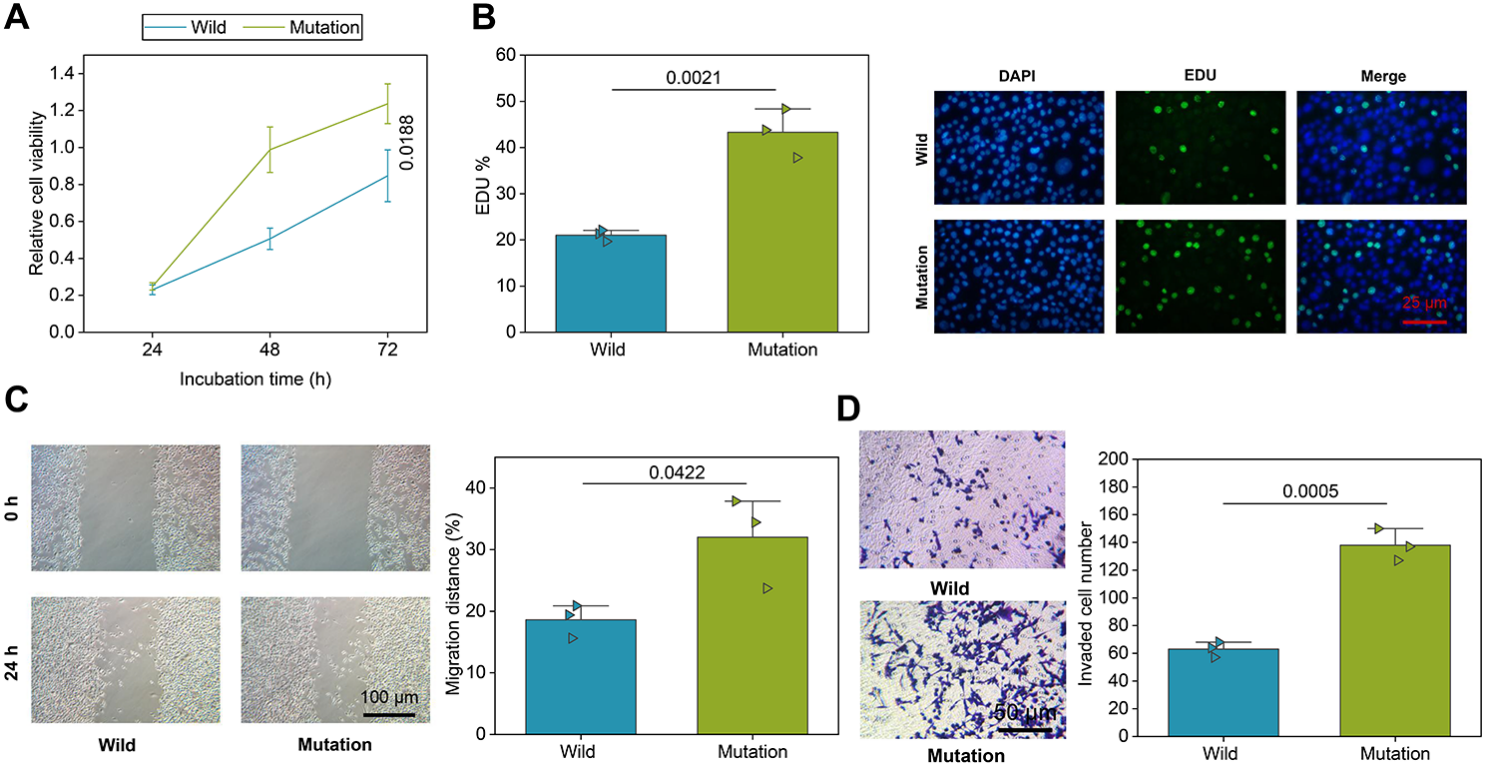
Effects of BAP1 mutations on the malignant phenotypes of UM cells. **A-B**, CCK-8 and EdU assays for proliferation of UM cells with WT or MUT BAP1. **C**, Scratch test for migration of UM cells with WT or MUT BAP1. D, Transwell assay for invasion of UM cells with WT or MUT BAP1. WT, wild type; mutated BAP1, mutant type BAP1. * *p* < 0.05

To further explore the effect of the BAP1 mutations on the malignant phenotypes of UM cells, we performed the CCK-8 and EdU assays. The results found that the UM cells with MUT BAP1 showed enhanced proliferation ability than those with WT BAP1 (Fig. 9A, B). Scratch test and Transwell assay revealed that the UM cells with MUT BAP1 had stronger migration and invasion abilities than those with WT BAP1. The above results suggested that the BAP1 mutations could result in enhancement of the malignant phenotypes of UM cells (Fig. 9C, D).

## Discussion

It has been demonstrated through genetic studies of UM that specific gene mutations may enable more accurate evaluation of metastatic risk of UM (Beasley et al. 2022). Herein, we set out to investigate whether BAP1 mutations could regulate the development in UM, and our results demonstrated that BAP1 mutations could induce the tumor immune microenvironment in UM by inactivating the NF-κB signaling pathway. A previous review discussed the contribution of inflammation to uveal melanoma and explored potential therapeutic targets related to the inflammatory tumor microenvironment, which have been identified and are currently being studied in preliminary phases with optimistic results (Liau et al. 2023). Angi et al. used proteomic profiling to examine the secretome of primary uveal melanoma tumors with high or low metastatic risk compared to normal choroidal melanocytes, identifying novel proteins and pathways that may contribute to metastatic development, particularly in the liver (Angi et al. 2016). The study by Jang et al. on primary uveal melanoma (pUM) utilized quantitative proteomic analysis to identify potential protein biomarkers for predicting metastasis and insights into UM metastasis, with a focus on identifying immune-related proteins that could be targeted for immune therapy checkpoint blockade (Jang et al. 2021).

Initially, our joint analysis through the TCGA and cBioPortal databases screened the top 10 genes with mutation frequency in UM samples. BAP1 mutations were further found to be closely related to the poor prognosis of UM patients and induce the immunosuppressive microenvironment in UM. Interestingly, BAP1 mutations were revealed to be associated with the immune infiltration and immune state of UM (Pan et al. 2020). Prior research also unraveled the correlation between BAP1 mutations and the metastasis and poor prognosis of UM (Baqai et al. 2022). In consistency with our results, a previous study found that deficiency of BAP1 expression was correlated with the immunosuppressive microenvironment in UM (Figueiredo et al. 2020). Our results also revealed that BAP1 mutations contributed to significant declines in the mRNA expression of BAP1 in UM cells. BAP1 mutations, which are mostly inactivated mutations, can affect BAP1 protein expression (Han et al. 2021; Kaler et al. 2022). The absence of BAP1 was observed in the UM cell lines, which was a feature of cancer aggression (Amirouchene-Angelozzi et al. 2014). Loss of BAP1 could promote the immunosuppressive microenvironment in Class 2 μm by regulating PROS1 (Kaler et al. 2022). These previous studies can support our finding that BAP1 mutations causing loss of BAP1 expression could induce the immunosuppressive microenvironment in UM.

Mechanistically, we demonstrated in the current study that BAP1 mutations could inhibit the NF-κB signaling pathway, thereby repressing the cytokine secretion and antigen presentation by macrophages and inducing immunosuppressive microenvironment in UM. Macrophages can alter the tumor microenvironment by producing cytokines (Huang and Yu 2020; Angi et al. 2016; Jang et al. 2021), and stimulation of the antigen presentation by macrophages may be promising for antitumor therapies (Muntjewerff et al. 2020). In this study, the aforementioned mechanism was verified using a co-culture system without in vivo animal experiments, for BAP1 would be inactivated after BAP1 mutation, and UM cell lines would not be metabolized in existing animal models; in addition, the current models mainly rely on immune-deficient mice, and if these mice were used, the immunosuppressive mechanism related to BAP1 mutations would not work (Kaler et al. 2022). Partially consistent with our results, BAP1 was unfolded to increase the COX-2 and mPGES-1 expression by activating the NF-κB signaling pathway and inducing the release of IL-1β (Viana et al. 2020), but this study failed to explore the regulatory role of BAP1 mutations on the NF-κB signaling pathway. Of note, the possible role of the NF-κB signaling pathway in the development of UM has been previously unveiled. For instance, NF-κB could be activated by NEMO, a downregulated gene in UM cells serving as a prognostic biomarker for UM (Singh et al. 2018). Besides, NF-κB-activated DCs could increase the release of cytokines including IL-12 and TNF-α to induce memory T-cell responses and facilitate MHCI and MHCII-restricted antigen presentation in UM (Koch et al. 2022). Cytokines secreted by macrophages can regulate the NF-κB signaling pathway, thereby promoting the transcription of immune regulators (Diep et al. 2022). NF-κB could stimulate active antigen presentation by macrophages (Altaf and Revell 2013), and activated NF-κB could induce MHC-I antigen presentation to potentiate cancer chemoimmunotherapy (Zhou et al. 2021). In our study, we revealed that the role of BAP1 mutations in the malignant phenotypes of UM cells and the growth and metastasis of UM was achieved by regulating the immunosuppressive microenvironment through inhibition of the NF-κB signaling pathway. However, there are conflicting findings in previous studies. In 2019, Zahra Souri et al. examined BAP1-negative UM cells using the Affymetrix Nsp array method and found elevated expression levels of NFkB1 and NFkB2, as well as decreased expression levels of SPP1 and PPARγ (Souri et al. 2019). Therefore, additional experimental validation is required to confirm the results of this study. Primary uveal melanoma (UM) originates from malignant transformation of melanocytes that originate from the neuroectoderm in the ciliary body. The liver is the most common site of metastasis when primary UM has migrated (Shain et al. 2019; Krishna et al. 2020). The tumor microenvironment of metastatic UM is different from that of primary UM. The mechanisms of metastatic UM are still unknown (Piquet et al. 2019; Babchia et al. 2019). This study focuses on the mechanisms of primary UM, and whether they are applicable to metastatic UM is still unknown. Therefore, our future research may be conducted to verify it.

## Conclusion

**Fig. 10:**
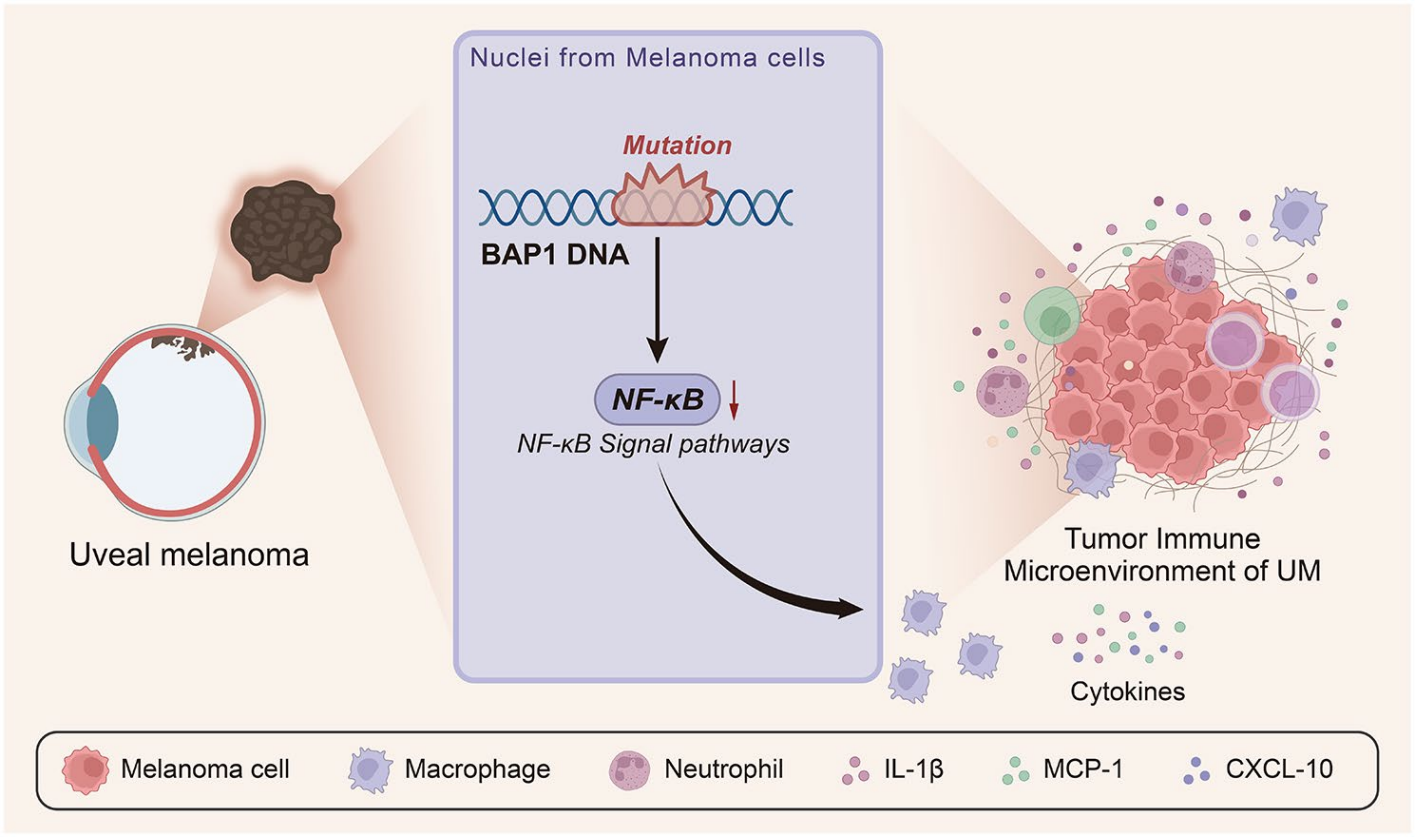
Schematic illustration of the molecular mechanism of BAP1 mutations affecting the tumor immune microenvironment in UM. BAP1 mutations may repress the NF-κB signaling pathway, repressing the cytokine secretion and antigen-presenting abilities of macrophages, which induces the immunosuppressive microenvironment, augments the malignant phenotypes of UM cells and ultimately promotes the growth and metastasis of UM.

A preliminary conclusion can be drawn in the current study that BAP1 mutations might repress the NF-κB signaling pathway, which repressed the cytokine secretion and antigen presentation by macrophages, inducing the immunosuppressive microenvironment, augmenting the malignant phenotypes of UM cells and ultimately promoting the growth and metastasis of UM (Fig. 10). This study may provide a new understanding of the mechanism of BAP1 mutations in UM.

### Electronic supplementary material

Below is the link to the electronic supplementary material.

Supplementary Material 1

## Data Availability

The data that supports the findings of this study are available on request from the corresponding author.

## Abbreviations

UM: Uveal melanoma
BAP1: BRCA1-associated protein 1
GSEA: Gene set enrichment analysis
NF-κB: Nuclear factor-κB
TMB: Tumor mutational burden
OS: Overall survival
PFS: Progression-free survival
MUT: Mutant type
WT: Wild type
NK: Natural killer
DEGs: Differentially expressed genes
