# Supplementary material for: BAP1 mutations inhibit the NF-κB signaling pathway to induce an immunosuppressive microenvironment in uveal melanoma
Source: Mol Med. 2023 Sep 14;29:126. doi: 10.1186/s10020-023-00713-7 (PMC10503157; doi:10.1186/s10020-023-00713-7)
Supplement: Supplementary file 1 — Supplementary Material 1 [file 10020_2023_713_MOESM1_ESM.docx]

**Table S1** CDS sequences for NF-κB

atggcagaagatgatccatatttgggaaggcctgaacaaatgtttcatttggatccttctttgactcatacaatatttaatccagaagtatttcaaccacagatggcactgccaacagatggcccataccttcaaatattagagcaacctaaacagagaggatttcgtttccgttatgtatgtgaaggcccatcccatggtggactacctggtgcctctagtgaaaagaacaagaagtcttaccctcaggtcaaaatctgcaactatgtgggaccagcaaaggttattgttcagttggtcacaaatggaaaaaatatccacctgcatgcccacagcctggtgggaaaacactgtgaggatgggatctgcactgtaactgctggacccaaggacatggtggtcggcttcgcaaacctgggtatacttcatgtgacaaagaaaaaagtatttgaaacactggaagcacgaatgacagaggcgtgtataaggggctataatcctggactcttggtgcaccctgaccttgcctatttgcaagcagaaggtggaggggaccggcagctgggagatcgggaaaaagagctaatccgccaagcagctctgcagcagaccaaggagatggacctcagcgtggtgcggctcatgtttacagcttttcttccggatagcactggcagcttcacaaggcgcctggaacccgtggtatcagacgccatctatgacagtaaagcccccaatgcatccaacttgaaaattgtaagaatggacaggacagctggatgtgtgactggaggggaggaaatttatcttctttgtgacaaagttcagaaagatgacatccagattcgattttatgaagaggaagaaaatggtggagtctgggaaggatttggagatttttcccccacagatgttcatagacaatttgccattgtcttcaaaactccaaagtataaagatattaatattacaaaaccagcctctgtgtttgtccagcttcggaggaaatctgacttggaaactagtgaaccaaaacctttcctctactatcctgaaatcaaagataaagaagaagtgcagaggaaacgtcagaagctcatgcccaatttttcggatagtttcggcggtggtagtggtgctggagctggaggcggaggcatgtttggtagtggcggtggaggagggggcactggaagtacaggtccagggtatagcttcccacactatggatttcctacttatggtgggattactttccatcctggaactactaaatctaatgctgggatgaagcatggaaccatggacactgaatctaaaaaggaccctgaaggttgtgacaaaagtgatgacaaaaacactgtaaacctctttgggaaagttattgaaaccacagagcaagatcaggagcccagcgaggccaccgttgggaatggtgaggtcactctaacgtatgcaacaggaacaaaagaagagagtgctggagttcaggataacctctttctagagaaggctatgcagcttgcaaagaggcatgccaatgcccttttcgactacgcggtgacaggagacgtgaagatgctgctggccgtccagcgccatctcactgctgtgcaggatgagaatggggacagtgtcttacacttagcaatcatccaccttcattctcaacttgtgagggatctactagaagtcacatctggtttgatttctgatgacattatcaacatgagaaatgatctgtaccagacgcccttgcacttggcagtgatcactaagcaggaagatgtggtggaggatttgctgagggctggggccgacctgagccttctggaccgcttgggtaactctgttttgcacctagctgccaaagaaggacatgataaagttctcagtatcttactcaagcacaaaaaggcagcactacttcttgaccaccccaacggggacggtctgaatgccattcatctagccatgatgagcaatagcctgccatgtttgctgctgctggtggccgctggggctgacgtcaatgctcaggagcagaagtccgggcgcacagcactgcacctggctgtggagcacgacaacatctcattggcaggctgcctgctcctggagggtgatgcccatgtggacagtactacctacgatggaaccacacccctgcatatagcagctgggagagggtccaccaggctggcagctcttctcaaagcagcaggagcagatcccctggtggagaactttgagcctctctatgacctggatgactcttgggaaaatgcaggagaggatgaaggagttgtgcctggaaccacgcctctagatatggccaccagctggcaggtatttgacatattaaatgggaaaccatatgagccagagtttacatctgatgatttactagcacaaggagacatgaaacagctggctgaagatgtgaagctgcagctgtataagttactagaaattcctgatccagacaaaaactgggctactctggcgcagaaattaggtctggggatacttaataatgccttccggctgagtcctgctccttccaaaacacttatggacaactatgaggtctctgggggtacagtcagagagctggtggaggccctgagacaaatgggctacaccgaagcaattgaagtgatccaggcagcctccagcccagtgaagaccacctctcaggcccactcgctgcctctctcgcctgcctccacaaggcagcaaatagacgagctccgagacagtgacagtgtctgcgacagcggcgtggagacatccttccgcaaactcagctttaccgagtctctgaccagtggtgcctcactgctaactctcaacaaaatgccccatgattatgggcaggaaggacctctagaaggcaaaatttag

**Table S2** Primer sequences for RT-qPCR

| Genes | Sequences |
| --- | --- |
| BAP1 (Human) | Forward: 5'-AGGAGCTGCTGGCACTGCTGA-3' |
|  | Reverse: 5'-TTGTGGAGCCGGCCGATGCT-3' |
| IL-1β (Human) | Forward: 5'-AGCTACGAATCTCCGACCAC -3' |
|  | Reverse: 5'-CGTTATCCCATGTGTCGAAGAA-3' |
| MCP-1 (Human) | Forward: 5'-CAGCCAGATGCAATCAATGCC-3' |
|  | Reverse: 5'-TGGAATCCTGAACCCACTTCT-3' |
| TGF-β1 (Human) | Forward: 5'-CTAATGGTGGAAACCCACAACG-3' |
|  | Reverse: 5'-TATCGCCAGGAATTGTTGCTG-3' |
| CXCL-10 (Human) | Forward: 5'-AGCAAGGAAAGGTCTAAAAGATCTCC-3′ |
|  | Reverse: 5'-GGCTTGACATATACTCCATGTAGGG-3′ |
| HLA-DRA (Human) | Forward: 5'-CCAGAGACTACAGAGAATGTG-3' |
|  | Reverse: 5'-TTGATGATGAAGATGGTCCCAA-3' |
| HLA-DRB (Human) | Forward: 5'-GAGCAGGTTAAACATGAGTGTC-3' |
|  | Reverse: 5'-CACGTACTCCTCTTGGTGATAG-3' |
| GAPDH (Human) | Forward: 5'-GCACCGTCAAGGCTGAGAAC-3' |
|  | Reverse: 5'-TGGTGAAGACGCCAGTGGA-3′ |

Note: RT-qPCR, reverse transcription quantitative real-time polymerase chain reaction; BAP1, BRCA1-associated protein 1; IL-1β, interleukin 1 beta; MCP-1, monocyte chemoattractant protein 1; TGF-β1, transforming growth factor beta 1; CXCL-10, C-X-C motif chemokine ligand 10; HLA-DRA, major histocompatibility complex, class II, DR alpha; HLA-DRB, major histocompatibility complex, class II, DR beta; GAPDH, glyceraldehyde 3-phosphate dehydrogenase.

**Table S3** Manufacturer information of primary antibodies

| Name | Cat. | Manufacturer | Country | Dilution ratio |
| --- | --- | --- | --- | --- |
| Rabbit anti-NF-κB | ab32360 | Abcam | USA | 1:1000-1:10000 |
| Mice anti-BAP1 | sc28383 | Santa Cruz Biotechnology | USA | 1:100-1:500 |
| Rabbit anti-mice IgG | ab6728 | Abcam | USA | 1:2000-1:10000 |
| Goat anti-rabbit IgG | ab6721 | Abcam | USA | 1:5000 |
| Rabbit anti-GAPDH | ab181602 | Abcam | USA | 1:10000 |

Note: BAP1, BRCA1-associated protein 1; NF-κB, nuclear factor-κB; IgG, immunoglobulin G; GAPDH, glyceraldehyde 3-phosphate dehydrogenase.
